# Lupus-Induced Myopic Shift

**DOI:** 10.7759/cureus.22961

**Published:** 2022-03-08

**Authors:** Sara Safari, Thomas A Weppelmann

**Affiliations:** 1 Department of Ophthalmology, University of South Florida (USF) Health Morsani College of Medicine, Tampa, USA

**Keywords:** periorbital edema, lupus nephritis, nephritis, systemic lupus erythematosus, myopic shift

## Abstract

While ophthalmic manifestations of lupus are common, a myopic shift is a rare manifestation of systemic lupus erythematosus (SLE). An acute myopic shift is defined as a progressive worsening of nearsighted vision within a short time frame. Here, we describe the unique presentation of a young woman with a lupus-induced acute myopic shift. The patient presented with blurry vision and bleeding gums with a previous abnormal lymph node biopsy to rule out ocular lymphoma or leukemia. Her baseline prescription prior to coming in was −4.0D in both eyes. Upon exam, she presented with vision worsening: −7.0D in the right eye and −8.2D in the left eye. After completing blood laboratory tests, it became clear that she had pancytopenia, kidney damage, and elevated inflammatory markers pointing towards lupus. A diagnosis of acute binocular myopic shift induced by systemic lupus erythematosus was made.

## Introduction

Systemic lupus erythematosus (SLE) is a chronic autoimmune inflammatory disease that can affect multiple organ systems. Under normal physiological conditions, apoptotic cells are phagocytosed. Apoptotic cellular content would normally trigger an inflammatory immune response if not phagocytosed [1]. SLE is associated with classical complement protein deficiency, which prevents the phagocytosis of immune complexes, apoptotic cell material, pathogens, and foreign material [2]. Therefore, because of the apoptotic cellular content that is released, lymphocytes target this content, leading to autoantibody creation. These autoantibodies include antinuclear antibodies (ANA), which are antibodies against the body's DNA and histones, and anti-double-stranded DNA antibodies (anti-dsDNA) [1]. As such, SLE involves antibody-antigen complex deposition in various tissues, which eventually leads to cell death and inflammation [1].

SLE is typically present in young post-pubescent females and occurs more commonly in patients of African American or Asian descent. The American College of Rheumatology’s list of common SLE symptoms includes rashes, photosensitivity, mouth sores, arthritis, cardiovascular inflammation, proteinuria, hematuria, abnormal blood tests with low blood cell counts, positive ANA, or anti-dsDNA antibodies [3]. SLE treatment typically includes antimalarial drugs to treat mouth sores, rashes, arthritis, and fatigue. Other SLE treatment options include immunosuppressants and prednisone to control inflammation [3].

One-third of patients with SLE present with ocular manifestations, which can be a marker for overall systemic disease activity and is associated with significant morbidity [4]. SLE can affect the optic nerve, eye, ocular adnexa, and periorbita. The most common ocular manifestation of SLE is keratoconjunctivitis sicca, which can lead to many corneal complications [4]. The majority of patients with SLE express at least one symptom of dry eye. Here, we describe the unique presentation of a young woman with a rare ophthalmic manifestation of acute myopic shift from SLE. An acute myopic shift is defined as a progressive worsening in nearsighted vision within a short time frame.

## Case presentation

A 17-year-old female of Vietnamese descent with a nearsighted vision that began eight years ago, her baseline prescription was −4.0D in both eyes, presented with sudden onset facial swelling, periorbital edema, and vision changes described as an inability to see objects up close. Before her visit, she received the second dose of the Pfizer coronavirus vaccine and experienced a low-grade fever. She also had facial swelling that persisted for two weeks, with worsening nearsighted vision. Her vision progressed from an inability to see a few feet in front of her to being unable to see less than one foot in front of her with glasses over five days. She also reported mouth ulcers, bleeding in her gums, and irritation while brushing her teeth for a week. The patient also had light sensitivity, a skin rash, headaches, and a history of migraines. Her blood laboratory values (Table 1) showed evidence of low complement proteins, pancytopenia, and nuclear antibodies. Urinalysis showed proteinuria and hematuria without acute kidney damage, which can also be seen in Table 1. Additionally, the patient had a past fine-needle aspiration biopsy in 2019 that revealed atypical lymphoid populations in a cervical lymph node; at that time, a lymphoproliferative disorder could not be ruled out. In 2019, there was a concern for possible Hodgkin's lymphoma; however, this was not followed upon. She also had mild protein-calorie malnutrition.

**Table 1:** Patient's abnormal blood and urine laboratory findings upon initial exam. ESR: erythrocyte sedimentation rate, ANA: antinuclear antibodies, WBC: white blood cell count, RBC: red blood cell count.

Laboratory findings	Patient values	Reference range
ESR (mm/hr)	57	0–20
Complement C3 protein (mg/dL)	17	83–193
Complement C4 protein (mg/dL)	<2.9	15–57
ANA	Positive	Negative
Anti-dsDNA antibody	Positive	Negative
WBC (10^3^/µL)	2.84	4.6–10.2
Hemoglobin (g/dL)	11.8	12.2–16.2
Total neutrophil count (10^3^/µL)	1.02	1.79–7.85
Platelets (10^3^/µL)	82	142.0–424.0
Urine protein (mg/dL)	100	negative
Urine RBC (RBC/high power field)	21–30	0–2

**Figure 1:**
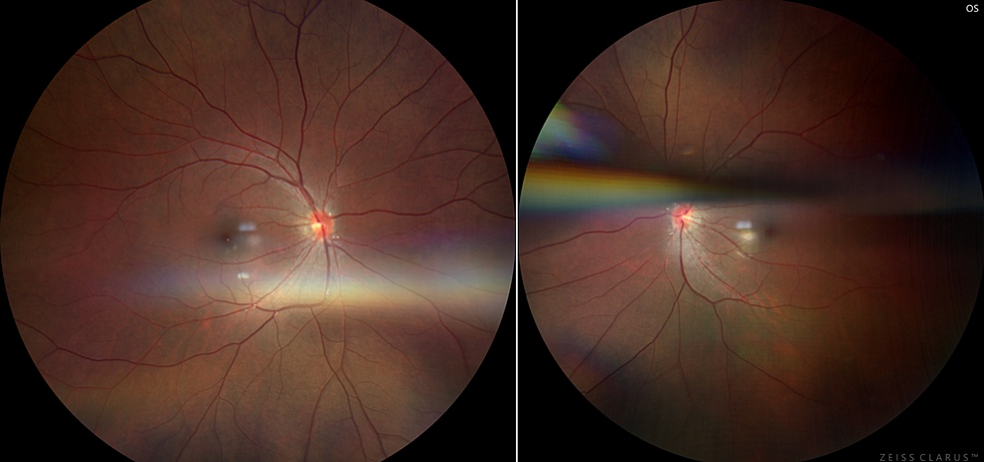
OD (left image) and OS (right image) color funduscopy images. Photos show clear vitreous, pink sharp and flat nerve, attached and dry macula, vessels without attenuation, and retina attached without pigment set changes. The OS image also includes an artifact. OD: right eye, OS: left eye.

**Figure 2:**
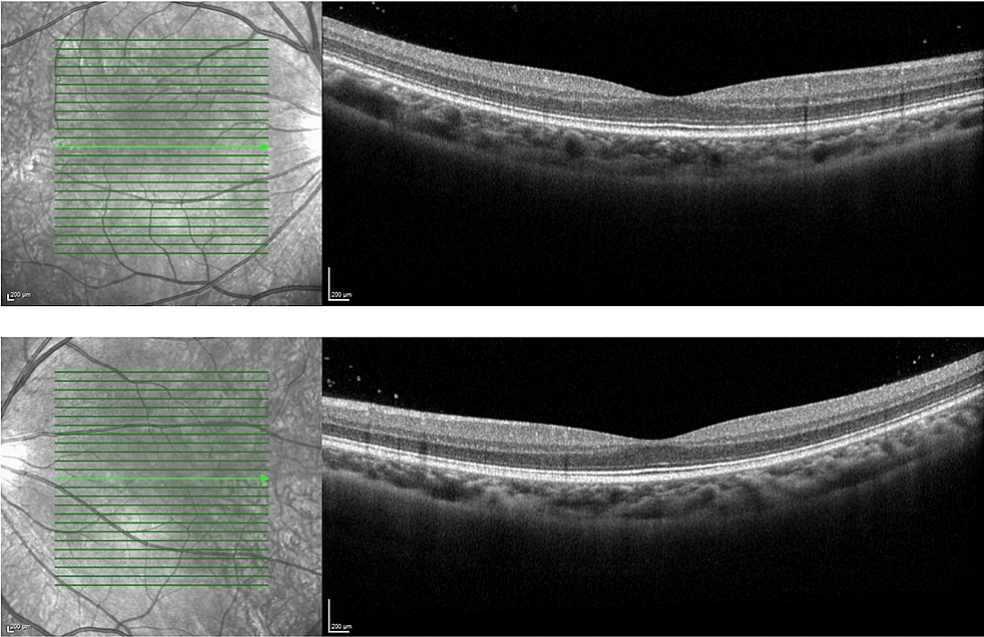
OD (image above) and OS (image below) OCT images showing the macula with normal retinal architecture without intraretinal or subretinal fluid. OD: right eye, OS: left eye, OCT: optical coherence tomography.

Her visual acuity, which can be appreciated in Table 2, showed worsening myopia with an inability to see faraway objects. Additionally, her intraocular pressures were 17 mmHg in both eyes. The patient's funduscopy images and optical coherence tomography (OCT) images can be appreciated in Figures 1 and 2, respectively. The rest of the eye exam was within normal limits, with a deep and quiet anterior chamber. Anterior chamber depth, lens thickness, and forward movement of the lens-iris diaphragm using ultrasound biomicroscopy were not done as it is not part of routine care. It was suspected that her vision loss was likely due to ciliary body inflammation.

**Table 2:** Patient's initial visual acuity exam findings. OD: right eye, OS: left eye, PH: pinhole refraction, Mrx: manifest refraction.

Eye	Visual acuity
OD	Far: 20/200-2, Far with Mrx (−7.00D), 20/40
	PH to 20/50-2 Near (+3.0 Runge): 20/32
OS	Far: 20/400, Far with Mrx (−8.20D), 20/50
	PH to 20/70-2 Near (+3.0 Runge): 20/50

In this patient with new-onset facial swelling, proteinuria, and hematuria in the setting of positive ANA and anti-dsDNA and low complement levels, systemic lupus erythematosus was suspected. Nephritis and proteinuria were the likely causes of her periorbital edema. With this patient's acute myopic shift over the last couple of days, lupus-induced myopic shift and uveitis syndrome were suspected in both eyes. Additionally, the patient was not on topiramate, sulfa drugs, diuretics, or other drugs known for causing myopic shift, thus ruling out these causes. We have no image of the patient's eyes because they were treated in the hospital.

From the ED, the patient was admitted for further work-up. She completed three days of pulse intravenous steroids and underwent a renal biopsy in the nephrology department. Her results indicated stage four lupus nephritis. Her diagnosis of SLE with other organ involvement was confirmed with kidney, skin, and hematologic involvement. She began immunosuppressive therapy treatment. Hydroxychloroquine, mycophenolate, sulfamethoxazole-trimethoprim, and famotidine (for gastric protection) were initiated, and prednisone was continued. The patient continued to come in for outpatient ophthalmology follow-ups for her lupus-induced myopic shift without uveitis, iridocyclitis, or optic neuritis, with no need for acute inpatient intervention. One month after treatment initiation, she was able to wear her previous glasses and contact lenses with a −4.0D prescription. She no longer had periorbital edema, redness, discharge, or strabismus. At her five-month follow-up appointment, her manifest refraction was −5.50 OD and −5.75 OS. Additionally, with her previous glasses, she presented with 20/20 corrected vision in both eyes.

## Discussion

Here, we provide a unique presentation of a patient with a lupus-induced acute myopic shift, which is a rare ocular manifestation of SLE. Our patient presented with rapidly worsening myopic vision and bleeding mouth ulcers. Upon blood laboratory analysis showing kidney damage and elevated inflammatory agents, further tests were done to confirm lupus. Once SLE was confirmed, a diagnosis of acute binocular myopic shift induced by lupus was made.

SLE can cause ocular manifestations through different mechanisms that include immune complex deposition, vasculitis, thrombosis, and other antibody-related mechanisms [5]. According to the literature, acute myopia can occur from changes in the lens curvature or anterior shift of the lens-iris diaphragm [6]. Anterior displacement occurs in ciliochoroidal effusion, ocular blunt trauma, and use of pilocarpine [6-8]. The acute myopic shift is also associated with ocular inflammation, ocular trauma, pregnancy, diabetes, and medications like topiramate [9].

There are numerous ocular manifestations of SLE that can be vision-threatening or a marker of systemic damage. Other reported cases of ocular SLE manifestations commonly include vision loss as a consequence [5]. Some forms of SLE-associated vision loss, like SLE retinopathy or ciliary body inflammation, can be treated by immunosuppressive therapy [9-11]. However, other forms of SLE-associated vision loss do not respond well to immunosuppressive therapy [5,10]. For example, studies have shown that macular infarction can cause rapid vision loss that cannot be resolved with SLE immunosuppressive therapy [10,11].

Other reported causes of lupus-induced myopic shift proposed uveal effusion with ciliary body swelling as the mechanism of vision loss [6,9,12]. Previous cases have shown ciliary body inflammation causing an anterior shift of the iris-lens diaphragm which led to a shallow anterior chamber. These cases responded well to immunosuppressive therapy [9,12]. The early diagnoses in these cases helped prevent further complications and promoted rapid condition improved after treatment. These findings are similar to our presented case, as our patient’s vision returned close to baseline after she began lupus treatment with immunosuppressive drugs; however, based on the slit-lamp exam, our patient’s anterior chamber was deep, not shallow. Ciliary body inflammation was initially hypothesized, but we hypothesize that a decrease in fluid dynamics from plasma incisive pressure could play a role in alterations to the natural lens, but the exact mechanism remains unknown.

## Conclusions

Ocular manifestations are common and, despite the rarity of myopic shift, they should be considered in patients experiencing vision loss with other SLE symptoms. This case also shows how quickly visual acuity can be restored to baseline following SLE treatment mechanisms like immunosuppressive therapy. Additionally, in our case, anterior chamber depth was only evaluated using a slit-lamp exam. Therefore, the patient's anterior chamber depth was not assessed using ultrasound biomicroscopy, which may be a current limitation. Ultrasound biomicroscopy is a test to be considered when making the diagnosis of a myopic shift since it can assess anterior chamber depth.

## References

[1] Mok CC, Lau CS (2003). Pathogenesis of systemic lupus erythematosus. J Clin Pathol.

[2] Janeway CA, Travers P, Walport M (2001). Immunobiology: The Immune System in Health and Disease. https://www.ncbi.nlm.nih.gov/books/NBK27100/.

[3] (2021). Lupus. https://www.rheumatology.org/I-Am-A/Patient-Caregiver/Diseases-Conditions/Lupus.

[4] Palejwala NV, Walia HS, Yeh S (2012). Ocular manifestations of systemic lupus erythematosus: a review of the literature. Autoimmune Dis.

[5] Sivaraj RR, Durrani OM, Denniston AK, Murray PI, Gordon C (2007). Ocular manifestations of systemic lupus erythematosus. Rheumatology (Oxford).

[6] Kamath YS, Singh A, Bhat SS, Sripathi H (2013). Acute onset myopia as a presenting feature of systemic lupus erythematosus. J Postgrad Med.

[7] Ikeda N, Ikeda T, Nomura C, Mimura O (2007). Ciliochoroidal effusion syndrome associated with posterior scleritis. Jpn J Ophthalmol.

[8] Bhattacharyya KB, Basu S (2005). Acute myopia induced by topiramate: report of a case and review of the literature. Neurol India.

[9] Yosar J, Whist E (2019). Acute myopic shift in a patient with systemic lupus erythematosus. Am J Ophthalmol Case Rep.

[10] Shein J, Shukla D, Reddy S, Yannuzzi LA, Cunningham ET Jr (2008). Macular infarction as a presenting sign of systemic lupus erythematosus. Retin Cases Brief Rep.

[11] Rao VA, Pandian DG, Kasturi N, Muthukrishanan V, Thappa DM (2010). A case to illustrate the role of ophthalmologist in systemic lupus erythematosus. Indian J Dermatol.

[12] Hung KC, Hsueh PY, Wang NK, Su WW, Tan HY (2011). Transient myopic shifting in systemic lupus erythematosus. Lupus.

